# Evolutionary Developments in Plant Specialized Metabolism, Exemplified by Two Transferase Families

**DOI:** 10.3389/fpls.2019.00794

**Published:** 2019-06-25

**Authors:** Hiroaki Kusano, Hao Li, Hiroshi Minami, Yoshihiro Kato, Homare Tabata, Kazufumi Yazaki

**Affiliations:** ^1^Laboratory of Plant Gene Expression, Research Institute for Sustainable Humanosphere, Kyoto University, Kyoto, Japan; ^2^Life Science Center, Hokkaido Mitsui Chemicals, Sunagawa, Japan

**Keywords:** prenyltransferase, acyltransferase, BAHD, *Citrus*, gene family, molecular evolution, specialized metabolism, *Lithospermum*, *Taxus*

## Abstract

Plant specialized metabolism emerged from the land colonization by ancient plants, becoming diversified along with plant evolution. To date, more than 1 million metabolites have been predicted to exist in the plant kingdom, and their metabolic processes have been revealed on the molecular level. Previous studies have reported that rates of evolution are greater for genes involved in plant specialized metabolism than in primary metabolism. This perspective introduces topics on the enigmatic molecular evolution of some plant specialized metabolic processes. Two transferase families, BAHD acyltransferases and aromatic prenyltransferases, which are involved in the biosynthesis of paclitaxel and meroterpenes, respectively, have shown apparent expansion. The latter family has been shown to beinvolved in the biosynthesis of a variety of aromatic substances, including prenylated coumarins in citrus plants and shikonin in *Lithospermum erythrorhizon*. These genes have evolved in the development of each special subfamily within the plant lineage. The broadness of substrate specificity and the exon-intron structure of their genes may provide hints to explain the evolutionary process underlying chemodiversity in plants.

## Introduction

Since land plant colonization 500 million years ago, plant specialized metabolic processes have expanded considerably, resulting in the development of diverse traits within the plant kingdom (Weng et al., 2012). The chemical diversity of those natural products provides various metabolites beneficial for human life, including compounds associated with flavor, color, taste, and medicine. A comparative genome analysis strongly suggested that gene duplications played a major role in the evolution of divergent metabolic pathways (Fani and Fondi, 2009). The increase in the number of gene copies may have allowed promiscuous diversity of the encoded enzymes, resulting in the synthesis of new metabolites and providing organismal fitness that enhances the establishment of biosynthetic pathways in the plant lineage. The expansion of plant specialized metabolism has been observed in the genome of *Selaginella moellendorffii*, a plant that diverged shortly after the establishment of vascular tissues in plant evolution (Banks et al., 2011). A representative example of these expanded gene families is cytochrome P450-dependent monooxygenases, which constitute 1% of the predicted proteome in *Selaginella*. The genome of liverwort, *Marchantia polymorpha*, also encodes many terpenoid biosynthetic enzymes sharing a common isoprenoid pathway, a derivative designated taxadiene for the synthesis of plant hormones like gibberellin (Bowman et al., 2017). In *Physcomitrella patens,* a diterpene *ent*-kaurene is converted to gibberellin-type diterpenes, which act as regulators of protonema differentiation (Hayashi et al., 2010).

Species of the gymnosperm *Taxus* synthesize unique diterpene compounds called “taxoids,” which include an important anticancer drug, paclitaxel, a derivative designated taxadiene (Guerra-Bubb et al., 2012). Over 350 taxoid compounds were identified by 1999, with these compounds having variable side residues at the C1, C2, C4, C5, C7, C9, C10, C13, and C14 positions of the core taxadiene skeleton (Baloglu and Kingston, 1999). Except for a partial biosynthetic route (Croteau et al., 2006), knowledge about the biosynthetic pathway of taxoids that contribute to the chemodiversity in *Taxus* is limited.

Because of their fine-tuned genome data resources, angiosperm species provide good model systems to study molecular mechanisms underlying the chemodiversity of plant metabolites (Kroymann, 2011). For example, meroterpenes, including furanocoumarin derivatives (Bourgaud et al., 2006) and shikonin derivatives that are lipophilic red naphthoquinone (Yazaki, 2017), are specialized metabolites synthesized through branched routes from a metabolic pathway common to the general phenylpropanoid and isoprenoid biosynthetic pathways (Yazaki et al., 2017). The term “primary metabolism” indicates processes required to sustain life, such as energy acquisition from glucose. These processes include, for example, the biosynthesis of ubiquinone, a component of the respiratory chain in mitochondria. The biosynthesis of shikonin derivatives involves steps common to those involved in ubiquinone biosynthesis. To avoid confusion in distinguishing between primary and specialized (secondary) metabolism, this article uses the term “common metabolism” rather than “primary metabolism” to indicate biosynthetic pathways conserved in a broad variety of organisms.

This perspective focuses on two enzyme families as examples of molecular evolutionary events: the aromatic substrate prenyltransferase family, which plays a key role in the diversification of phenolics, and the BAHD (BEAT-AHCT-HCBT-DAT; initials of representative members) acyltransferase family, which is responsible for the derivatization of a core metabolite.

## Evolution of the *Citrus* Prenyltransferase Gene Family

Among prenyltransferase superfamily including prenyl chain elogation enzymes, aromatic prenyltransferases represent a family responsible for the prenylation of aromatic substances. An aromatic prenyltransferase of *Citrus limon*, ClPT1, is responsible for the biosynthesis of 8-geranylumbelliferone, a coumarin derivative of a plant specialized metabolite (Munakata et al., 2014). The chemical diversity of coumarin derivatives is greatly increased by the involvement of aromatic prenyltransferases, which have been identified in many plant lineages during the last decade (Karamat et al., 2014; Munakata et al., 2014). Phylogenetic analysis has suggested that the diverse prenyltransferases developed independently in each plant family rather than developing from a common ancestor within the prenyltransferase gene family (Munakata et al., 2016). The plant prenyltransferase gene family contains conserved subfamilies responsible for the ubiquinone, plastoquinone, and vitamin E biosynthesis pathways (Li, 2016).

**Figure 1:**
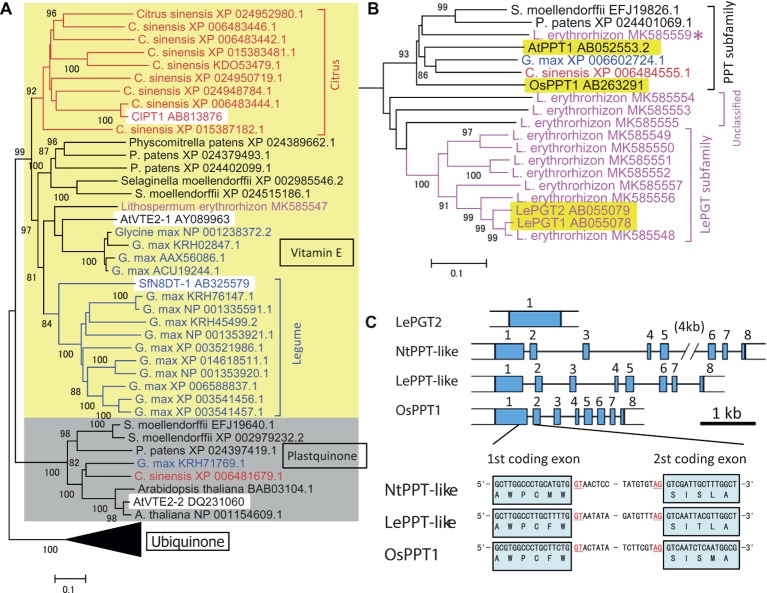
Phylogenetic analysis of the prenyltransferase family in citrus species and *Lithospermum erythrorhizon* as well as in model plants. **(A)** Grouping of plant prenyltransferases into three major clades: a clade represented by the *Arabidopsis* homogentisate phytyltransferase AtVTE2-1 involved in vitamin E biosynthesis (indicated by “Vitamin E” and a yellow background), a clade represented by *Arabidopsis* AtVTE2-2 for plastoquinone biosynthesis (indicated by “Plastoquinone” and a gray background), and a clade represented by the rice polyprenyltransferase OsPPT1 for ubiquinone biosynthesis (indicated by “Ubiquinone” and a compressed black triangle). Biochemically characterized proteins are indicated by a white background. The *Citrus* and legume proteins are shown in red and blue letters, respectively, and the lineage-specific clades are indicated by brackets with the same colors. **(B)** Details of the phylogenetic tree of polyprenyltransferases for ubiquinone in panel **(A)**. *L. erythrorhizon* proteins are shown in magenta letters. The brackets indicate subclades of polyprenyltransferases involved in ubiquinone biosynthesis (PPT subfamily), LePGT-like proteins (LePGT subfamily), and unclassified subclade proteins (unclassified). The proteins from other organisms are shown in black letters. The asterisk indicates the putative PPT-like protein of *L. erythrorhizon*. The phylogenetic tree was drawn using the MEGA7 neighbor-joining method with 1,000 bootstrap replicates for alignment of polyprenyltransferase-related proteins, which were calculated with the MUSCLE algorithm. The accession numbers are shown next to the name of the organism. Biochemically characterized proteins are indicated by a yellow background. The scale bar represents 0.1 amino acid substitutions per site. **(C)** LePGT gene is encoded by a single exon gene in the *L. erythrorhizon* genome, whereas LePPT-like proteins are encoded by genes with inserted introns, at positions similar to those of the authorized OsPPT gene and the closest tobacco homolog, NtPPT-like gene (gene=LOC107804153). The first intron insertion site into the coding region is shown. Scale bar, 1kb of DNA sequence. Blue boxes represent coding exons.

An outline of the evolutionary development of plant aromatic prenyltransferases in *Citrus* species was revealed by a phylogenetic analysis of previously characterized prenyltransferases and prenyltransferases of the model species *P. patens, S. moellendorffii, Arabidopsis thaliana, Glycine max,* and *Lithospermum erythrorhizon* (see below), in addition to *Citrus sinensis* (Figure 1A). Phylogenetically, these intrinsic membrane proteins can begrouped into three major subfamilies, i.e., those involved in the biosynthesis of vitamin E, plastoquinone, and ubiquinone (shown as yellow and gray backgrounds and as the black triangle, respectively in Figure 1A, with the black triangle expanded in Figure 1B). The biochemical functions of AtVTE2-1 (Savidge et al., 2002), AtVTE2–2 (Venkatesh et al., 2006), and OsPPT1 (Ohara et al., 2006) have been described. As expected from their fundamental roles, all model plant species had one or more proteins in each subfamily. In contrast, a search of the *C. sinensis* database revealed nine prenyltransferase-like proteins, forming a *Citrus*-specific subfamily within the vitamin E clade (shown in red in Figure 1A). A similar result was obtained by searching *Citrus clementina* genome sequences. These results suggest that *Citrus* species have developed a unique, expanded gene subfamily for specialized metabolism, with ClPT1 being biochemically characterized. This analysis also identified a similar unique subfamily expansion in *G. max* (shown in blue in Figure 1A). The first flavonoid-specific prenyltransferase SfN8DT1 from a legume species *Sophora flavescens* (Sasaki et al., 2008) is in this group, suggesting that flavonoid prenyltransferases in soybeans were derived from a vitamin E biosynthetic enzyme. Other, later detected flavonoid prenyltransferases were all classified in this subgroup (Akashi et al., 2009; Yoneyama et al., 2016). Most of these enzymes involved in specialized metabolism show strict substrate specificity in relation to a particular prenyl diphosphate.

Prenyltransferases involved in common metabolism show broad specificity in relation to substrates of different side chain lengths; i.e., they accept various prenyl diphosphates of different chain lengths (Sadre et al., 2010). For example, the ubiquinone biosynthesis pathway in rice can bemodified by introducing a decaprenyl diphosphate synthase, resulting in the production of non-native UQ10 rather than native UQ9 (Ohara et al., 2006; Takahashi et al., 2006). These expanded gene families and the broad substrate specificity of prenyltransferases may provide the opportunity for neo-functionalization of new enzymes in plant evolutionary history.

## Evolution of the *p*-hydroxybenzoic Acid Geranyltransferase Gene for Shikonin Biosynthesis

A boraginaceaeous medicinal plant, *L. erythrorhizon,* possesses a unique subfamily of *p*-hydroxybenzoic acid geranyltransferases (PGTs) (Figure 1B) that are specifically involved in shikonin biosynthesis (Yazaki et al., 2002). An overview of the evolutionary history of PGT was attained by assessing genome sequences and transcriptomes of *L. erythrorhizon* from the GenBank datasets SRP108575 and SRP141330, respectively, as well as by reassembling our original data (Takanashi et al., 2019). The hypothetical PGT-like proteins were found to beclosely related to the ubiquinone prenyltransferase subfamily involved in common metabolism (magenta in Figure 1B), which was closer to these hypothetical PGT-like proteins than the specialized citrus prenyltransferases (Figure 1A). Most PGT-like proteins are encoded by genes with a single exon, whereas general ubiquinone biosynthetic polyprenyltransferases (PPTs) are encoded by genes containing multiple exons (Figure 1C). It is of interest to determine how the single exon structure was generated during the evolution of plant specialized metabolism.

## Missing Ubiquinone Prenyltransferase in *L. erythrorhizon*

Although ubiquinone is a common metabolite in all eukaryotes, and the genes encoding PPTs are essential for the survival of a broad range of organisms, no orthologous ubiquinone PPT gene was found in the *L. erythrorhizon* transcriptome. Experiments in yeast showed that LePGT cannot synthesize ubiquinone (Yazaki et al., 2002), and LePGT heterologously expressed in *E. coli* was found to inhibit ubiquinone biosynthesis (Wu et al., 2015). Genomic sequencing identified a contig fragment that could code for PPT-like proteins (asterisk in Figure 1B) and that its amino acid sequence was moderately similar to that of OsPPT1, which is responsible for ubiquinone biosynthesis in rice. In addition, there were three contigs that wecould not classify, which are labeled “unclassified genes” (“unclassified” in Figure 1B). In contrast to the particular PGT that catalyzes shikonin biosynthesis, an intron insertion was found in the hypothetical gene, at the same position as in the PGTs of *Nicotiana tabacum* and *Oryza sativa* (Figure 1C). This conserved exon-intron organization was also observed in the PPT genes from *Arabidopsis* and rice (Ohara et al., 2006). This gene product is a strong candidate for a ubiquinone prenyltransferase in *L. erythrorhizon*, and its biochemical characterization is expected in the future.

## Evolution of the *Taxus* Acyltransferase Gene Family

Acyltransferases also substantially contribute to the diversification of specialized metabolites, in which BAHD and SCPL (serine carboxypeptidase-like) are representatives. Taxoids such as paclitaxel present in *Taxus* species are specialized metabolites and highly acylated compounds. Five known taxoid acyltransferases are closely related to each other, with all grouped in clade V of the BAHD acyltransferase family (D’Auria, 2006). These *Taxus* proteins differ in substrate specificities for both acyl donors and acceptors; i.e., they can utilize acetyl-CoA, benzoyl-CoA or phenylalanoyl-CoA for *O*- and *N*-acylation of various taxoid molecules (D’Auria, 2006).

**Figure 2:**
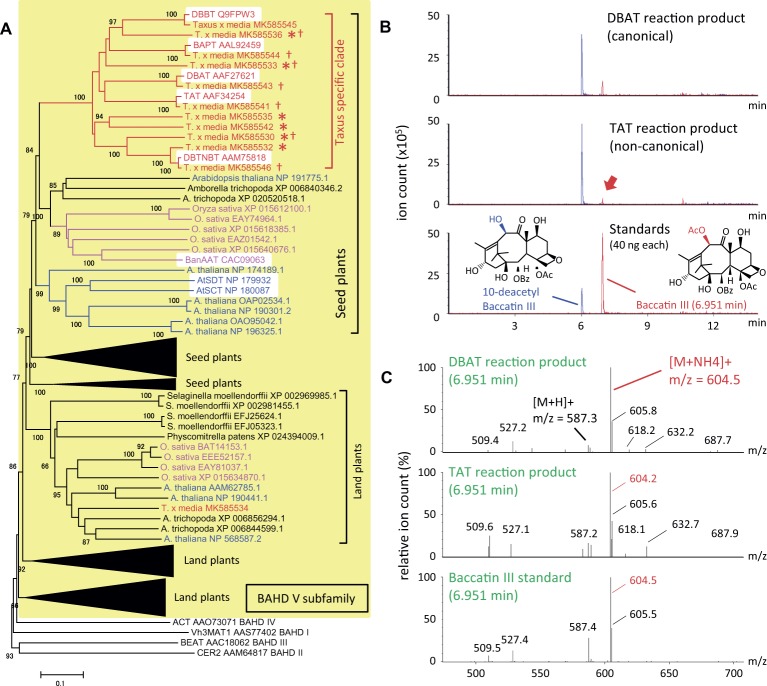
Phylogenetic analysis of BAHD acyltransferase proteins from *Taxus* species and LC-MS/MS analysis of the reaction products of the noncanonical enzyme, taxadienol 5-acyltransferase. **(A)** Performance of phylogenetic analysis with hypothetical *Taxus* BAHD acyltransferase-like proteins and related proteins from model plant species. The BAHD family was classified into five clades (D’Auria, 2006), with clade V indicated by a yellow background, and representatives of clade I–IV (Vh3MAT1, CER2, BEAT, and ACT, respectively) placed outside the yellow background. Proteins of *Taxus*, rice, *Arabidopsis* are shown in red, magenta, and blue letters, respectively, and the lineage-specific subclades are indicated by the same colors. The bracket “Taxus specific clade” indicates the *Taxus* lineage-specific subclade containing the five characterized proteins, TAT, DBAT, DBTNBT, DBBT, and BAPT, indicated by a white background. Asterisks indicate *Taxus* proteins of unknown function, and daggers indicate proteins biochemically analyzed in the present study. A representative widely conserved clade in land plants from *Physcomitrella* to *Arabidopsis* is indicated by brackets, with four other subclades compressed (expanded in Supplementary Figure S1), in addition to the clade conserved in seed plants containing the *Taxus* specific clade. The accession numbers are given next to the organism names. The phylogenetic tree was drawn using the MEGA7 neighbor-joining method with 1,000 bootstrap replicates for alignment calculated with the MUSCLE algorithm. Scale bar, 0.1 amino acid substitutions per site. **(B)** LC-MS/MS chromatograms of the enzyme reaction products of *Taxus* acyltransferases DBAT and TAT using acetyl-CoA and 10-DAB as substrates. The red arrow indicates the peak of the noncanonical reaction product. The bottom panel shows the chromatogram of standard specimens, 10-DAB and baccatin III. The chromatograms show a trace of representative ions m/z=545.5 [M+H]+and 604.5 [M+NH4]+for the substrate 10-DAB (blue) and the product baccatin III (red), respectively. The vertical axis indicates the value relative to 5 million ion counts. **(C)** Mass spectrum of the *in vitro* reaction product peaks found at a retention time of 6.951min of the chromatogram. The vertical axis indicates the relative value of ion count of maximum signal at m/z=604.5. The molecular formulas of 10-DAB and baccatin III are shown in panel **(B)**.

To understand the evolutionary development of the *Taxus* BAHD acyltransferase family, BAHD clade V was analyzed phylogenetically in detail (yellow background in Figure 2A). The amino acid sequences of *Taxus* BAHD members were obtained from the transcriptome data of *Taxus x media* cultured cells (Yukimune et al., 1996). Phylogenetic analysis showed that the *Taxus* BAHD proteins form a *Taxus*-specific clade (red bracket in Figure 2A), containing all five characterized acyltransferases (white background in the *Taxus*-specific clade), as well as other *Taxus* proteins of unknown function (asterisk in Figure 2A). Within this clade of the BAHD family, *O. sativa* and *A. thaliana* each form a unique clade, suggesting that lineage-specific subfamily expansion of the BAHD acyltransferases plays a major role in plant evolution (Fani and Fondi, 2009). In addition to this *Taxus*-specific subgroup, other *Taxus* BAHD proteins have been identified, with these classified with other model plant BAHD members (Supplementary Figure S1), suggesting that *Taxus* species possess genes encoding general BAHD clade V proteins that are conserved among a broad range of plant species.

It can behypothesized that neo-functionalization is induced by the acquisition of promiscuous enzymatic activity during plant evolution. Wehave examined the enzymatic activity of recombinant proteins prepared from seven isolated cDNAs encoding BAHD members of the *Taxus*-specific subfamily (dagger in Figure 2A). Each crude recombinant enzyme was prepared using pET22a and OrigamiB as a host-vector system (Novagen), without a periplasmic signal sequence, according to the conventional method. Each enzyme was reacted with acetyl-CoA and 10-deacetyl baccatin III (10-DAB) as substrates, and the reaction products were analyzed using an UPLC–MS/MS system equipped with a BEH C18 column (Waters). The clone encoding 5-hydroxytaxadiene 5-*O*-acetyltransferase (TAT) had 10-DAB:10-*O*-acetyltransferase (DBAT) activity (Walker et al., 2000), as well as the canonical enzyme DBAT (Figures 2B,C; Walker and Croteau, 2000). The amount of the product formed by the substrate was 1.4mol% for TAT and 10.4% for DBAT, suggesting that the activity of TAT was 13.2% that of DBAT. This promiscuity of enzymatic activity may represent the evolutionary footprint of a biosynthetic enzyme that acquires a new functionality through the alteration of substrate and product specificities, resulting in the production of a unique specialized metabolite.

## Conclusions and Perspectives

Using two transferase subfamilies as examples, wehave shown the “heritage” of expansion of a gene family, which is relevant for the development of plant specialized metabolic pathways. A protein in the specific BAHD subfamily of *Taxus* species showed promiscuous enzymatic activity for noncanonical substrates containing side chains at a noncanonical carbon position. These observations fit the general context of developmental molecular evolution that explains the development and establishment of new canonical enzymatic activity (Weng et al., 2012). The generation in *L. erythrorhizon* of a PGT gene subfamily, each containing a single exon and involved in shikonin biosynthesis, suggests the putative involvement of the reverse transcription of mature mRNA. If this surmise is valid for other enzyme families, single exon genes may provide clues to identifying missing proteins responsible for biosynthetic pathways for other valuable plant specialized metabolites.

There are yet many missing links, even in actively studied shikonin and taxoid biosynthetic pathways. The applicable range of the single exon hypothesis may not belimited only to biosynthetic enzymes, but to regulatory factors. The identification of regulatory factors will beessential to understanding the production of plant specialized metabolites, including membrane transporters. Comparative genomics will enable the assessment of the evolutionary footprint of these genes, e.g., the expansion of specific subfamilies and the proliferation of single exon genes. Further biochemical and molecular genetics studies may provide experimental evidence for the involvement of hypothetical proteins in plant specialized metabolism.

## Data Availability

The datasets generated for this study can befound in GenBank.

## Author Contributions

HK and KY wrote the manuscript and performed the phylogenetic and biochemical analyses. HL was involved in the assembly of genomic contigs and the analysis of the exon-intron structure of *Lithospermum erythrorhizon* genes. HM, YK, and HT were responsible for transcriptome analysis of *Taxus* spp.

### Conflict of Interest Statement

The authors declare that the research was conducted in the absence of any commercial or financial relationships that could beconstrued as a potential conflict of interest.

## Supplementary Material

The Supplementary Material for this article can befound online at: https://www.frontiersin.org/articles/10.3389/fpls.2019.00794/full#supplementary-material

FIGURE S1 Expanded phylogenetic tree of Figure 2A. Phylogenetic analysis with hypothetical Taxus BAHD acyltransferase-like proteins and related proteins from model plant species. Asterisks indicate Taxus proteins found in this study. Functionally identified BAHD proteins are highlighted in yellow background.
